# Pericardial Adhesions of Tuberculous Nature and Cirrhosis of the Liver in Children

**Published:** 1909-08

**Authors:** 


PERICARDIAL ADHESIONS OF TUBERCULOUS NATURE AND CIRRHOSIS OF THE LIVER IN CHILDREN.—Hutinel describes a somewhat rare condition in which a tuberculosis of the bronchial and mediastinal glands is communicated to the pericardium, producing adhesive pericarditis. Later cirrhosis of the liver follows, accompanied by ascites, and a fatal issue from heart failure ends the case. He calls it cardio-tuberculous cirrhosis. It begins sometimes insidiously with bronchitis and wasting. At other times it is more acute and begins with a pleurisy. Hypertrophy of the liver follows, the consistency of the organ being firm. Then appear circulatory troubles. The lips become cyanotic and the face violet, as in congenital heart trouble. There is dyspnoea, but no cardiac signs can be elicited. The urine is abundant and albuminous. The tuberculous condition has remained latent, and it is the liver signs that attract all attention. The pleura and pericardium are adherent, and the heart apex does not move with the beat. Treatment has so far failed to prevent a fatal issue.—*Le Bulletin Médical.*

